# Relationship between Bell’s Palsy and Previous Statin Use: A Case/Non-Case Study

**DOI:** 10.3390/ijerph17228408

**Published:** 2020-11-13

**Authors:** So Young Kim, Jee Hye Wee, Chanyang Min, Dae-Myoung Yoo, Hyo Geun Choi

**Affiliations:** 1 Department of Otorhinolaryngology-Head & Neck Surgery, CHA Bundang Medical Center, CHA University, Seongnam 13496, Korea; sossi81@hanmail.net; 2 Department of Otorhinolaryngology-Head & Neck Surgery, Hallym University College of Medicine, 22, Gwanpyeong-ro 170beon-gil, Dongan-gu, Anyang-si, Gyeonggi-do, Anyang 14068, Korea; weejh07@hanmail.net; 3 Hallym Data Science Laboratory, Hallym University College of Medicine, Anyang 14068, Korea; joicemin@naver.com (C.M.); ydm1285@naver.com (D.-M.Y.); 4 Graduate School of Public Health, Seoul National University, Seoul 08826, Korea

**Keywords:** Bell’s palsy, hydroxymethylglutaryl-CoA reductase inhibitor, cohort study, case/non-case Bell’s palsy study

## Abstract

We intended to determine the relationship between previous statin use and Bell’s palsy in a large study population receiving statins for the past 2 years. The Korean National Health Insurance Service—Health Screening Cohort data from 2002 to 2015 were collected. Participants with Bell’s palsy (*n* = 3203) were matched with participants without Bell’s palsy (*n* = 12,812). The number of days of previous statin use for 2 years before the onset of Bell’s palsy was analyzed using conditional logistic regression. Subgroups of age, sex, obesity, smoking, alcohol consumption, total cholesterol, and blood pressure were analyzed for any association between Bell’s palsy and prior statin use. The Bell’s palsy group reported greater statin use than the non-Bell’s palsy group (84.6 (standard deviation, SD = 201.7) vs. 74.4(SD = 189.4), *p* = 0.009). Previous statin use was associated with Bell’s palsy in the crude model (95% confidence intervals = 1.03–1.19, *p* = 0.006). However, this relationship disappeared when the possible covariates were adjusted for in model 2. All subgroups showed no increased odds for Bell’s palsy in previous statin users. We did not find an association between Bell’s palsy and previous statin use in this Korean population aged ≥40 years.

## 1. Introduction

3-Hydroxy-3-methylglutaryl coenzyme A reductase inhibitors, often referred to as statins, are commonly prescribed for dyslipidemia treatment [1]. A growing number of studies have independently reported multiple effects of statins regarding the reduction of low-density lipoprotein cholesterol [2,3]. Statins inhibit the synthesis of isoprenoids and the prenylation of small GTP-binding proteins, which in turn alter the expression of cascade molecules, including endothelial nitric oxide synthases, pro-inflammatory cytokines, and reactive oxygen species [2]. Therefore, statin treatment has been reported to be effective for many cardiovascular diseases, such as coronary artery disease [4,5], arrhythmia [5], and stroke [5,6], and non-cardiovascular diseases, such as Alzheimer’s disease [7], Parkinson’s disease [8], spinal cord injury [9], and infectious diseases [10,11]. Statin use has also been reported to have protective or therapeutic effects on peripheral neuropathy [12,13,14]. However, there have been a few conflicting results. A few previous studies suggest a relationship between prolonged statin use and an increased risk of peripheral neuropathy [15,16].

Bell’s palsy is characterized as an acute peripheral facial palsy or paresis without known organic causes (idiopathic) [17]. The prevalence of Bell’s palsy is estimated to be approximately 11–40/100,000 persons [18,19]. Reportedly, the peak of Bell’s palsy onset is observed at middle-age (between 39 and 50 years) [20]. Although the cause is elusive, viral factors, autoimmunity, and inflammatory changes of facial nerves are the proposed mechanisms of Bell’s palsy [21,22,23]. A health claim cohort study reported increased risk of Bell’s palsy with previous statin use (adjusted odds ratio (OR) = 1.47, 95% confidence interval (CI) = 1.28–1.69) [24]. The study suggested that statins mediate the risk of Bell’s palsy by impeding neural growth and maintenance, modulating immune responses, and triggering autoimmunity [24]. However, previous experimental and clinical studies have reported neuroprotective and anti-inflammatory effects of statins, indicating that statins might have protective roles against peripheral neuropathy [2,12,13,14].

We hypothesized that the positive relationship between Bell’s palsy and statin use might have been overestimated in the previous study. To assess the impact of statin use on Bell’s palsy, a longer period of statin use was selected (2 years). In addition, a matched non-Bell’s palsy group was compared with a Bell’s palsy group to determine any association with previous statin use. Past medical history, life-style factors, and laboratory measures were adjusted to alleviate possible confounder effects.

## 2. Materials and Methods

### 2.1. Study Population and Participant Selection

The ethics committee of Hallym University approved the present study (23 October 2019) and waived the requirement for written informed consent. The Korean National Health Insurance Service–Health Screening Cohort (NHIS-HEALS) data were used in the study (Supplementary S1 description) [25]. In brief, all Koreans ≥40 years old receive biannual health checks without cost. Because all Koreans are registered in the NHIS, all health insurance claim codes, diagnostic codes, death records, socio-economic data, and health screening data could be acquired for the present study.

**Figure 1:**
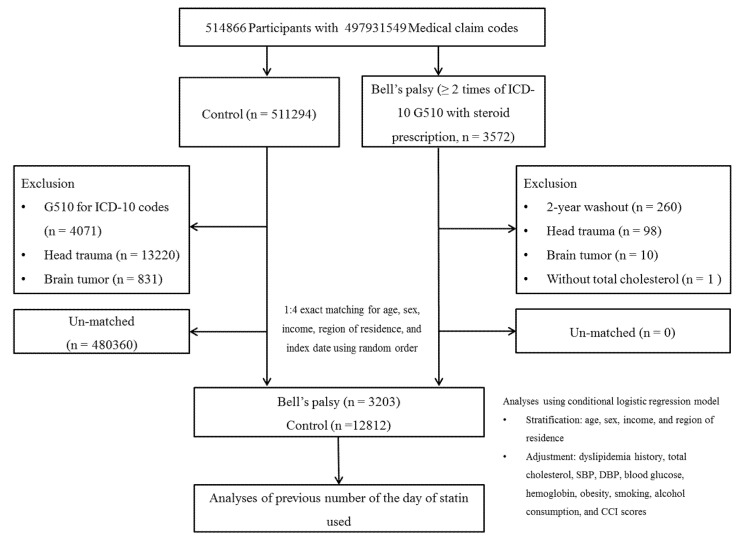
A schematic illustration of the participant selection process used in the present study. Of a total of 514,866 participants, 3185 with Bell’s palsy were matched with 12,740 individuals without Bell’s palsy for age, sex, income, and region of residence.

From a total of 514,866 participants with 615,488,428 medical claim codes, 3572 patients with Bell’s palsy were enrolled. Among the total participants, those without Bell’s palsy were included in the non-Bell’s palsy group (*n* = 511,294). Participants who were treated for the ICD-10 code G510 (Bell’s palsy) from 2002 to 2015 were excluded from the non-Bell’s palsy group (*n* = 4071). Patients who were first diagnosed with this condition between 2002 and 2003 were excluded from the Bell’s palsy group (*n* = 260). Participants who were previously diagnosed with head trauma (ICD-10 codes S00 to S09) two or more times and underwent head and neck computed tomography evaluations (claim codes: HA401-HA416, HA441-HA443, HA451-HA453, HA461-HA463, or HA471-HA473) were excluded (*n* = 98 from the Bell’s palsy group, *n* = 13,220 from the non-Bell’s palsy group). Participants with brain tumors (ICD-10 codes C70 to C72) two or more times were also excluded (*n* = 10 from the Bell’s palsy group, *n* = 831 from the non-Bell’s palsy group). One participant from the Bell’s palsy group was excluded owing to missing data on total cholesterol levels. The remaining 480,360 participants in the non-Bell’s palsy group were enrolled for matching. The Bell’s palsy group was matched (1:4) with randomly chosen non-Bell’s palsy participants for age, sex, income, and region of residence. Finally, 3203 patients with Bell’s palsy and 12,812 non-Bell’s palsy participants were included (Figure 1). The initial diagnosed date of Bell’s palsy was defined as the index date of each patient, and this date was identical between the Bell’s palsy and non-Bell’s palsy participants.

### 2.2. Previous Statin Use (Exposure)

The number of days of statin use was followed for 2 years before the index date.

### 2.3. Bell’s Palsy (Outcome)

Bell’s palsy was classified based on its ICD-10 code (G510). Patients with Bell’s palsy with a history of two or more treatment events and those who were prescribed steroids were selected, according to our previous studies [26,27].

### 2.4. Covariates

Age groups were categorized with five-year intervals. Income groups were classified into five levels. The regions of residence were divided into urban and rural areas [26,27].

Data on tobacco smoking, alcohol consumption, and body mass index (kg/m^2^) groups were collected, as previously described (Supplementary S1 description) [26,27]. Charlson comorbidity index (CCI) was included in the analysis [28,29].

We used the previous total cholesterol (mg/dL), systolic blood pressure (mmHg), diastolic blood pressure (mmHg), blood glucose (mg/dL), and hemoglobin (g/dL) measured on the health check-up date before the index date. Dyslipidemia was confirmed if participants were treated according to the ICD-10 code (E78) before the index date. For accuracy of diagnosis, dyslipidemia history was assigned to patients with a history of two or more treatment events.

### 2.5. Statistical Analyses

The Bell’s palsy group was compared with the non-Bell’s palsy group using Chi-square test for categorical variables and independent t test for continuous variables. The ORs and 95% CIs for statin use per year were calculated for patients with Bell’s palsy by conditional logistic regression. A crude model, model 1 (adjusted for dyslipidemia history, total cholesterol, systolic blood pressure, diastolic blood pressure, blood glucose, and hemoglobin), and model 2 (adjusted for model 1 plus obesity, smoking, alcohol consumption, and CCI score) were analyzed. The age, sex, income, and region of residence of the participants were stratified. Age (<60 years and ≥60 years), sex (males and females), and obesity status (underweight, normal weight, overweight, and obese) were sub-grouped and analyzed.

Two-tailed analyses were performed, and results with *p* values less than 0.05 were considered significant. The analyses were performed using SAS version 9.4 (SAS Institute Inc., Cary, NC, USA).

## 3. Results

The number of days of statin use was higher in the Bell’s palsy group (84.6 (standard deviation, SD = 201.7) vs. 74.4 (SD = 189.4), *p* = 0.009) (Table 1). In the Bell’s palsy group, 22.6% (725/3203) of participants had used statins previously; however, in the non-Bell’s palsy group, 20.2% (2591/12,812) of participants had previously used statins. The number of patients with dyslipidemia history was higher in the Bell’s palsy group (34.7% (1112/3203) vs. 29.6% (3794/12,812), *p* = 0.009). Total cholesterol, SBP, DBP, blood glucose, and hemoglobin levels were different between the groups. The distributions of obesity, alcohol consumption, and CCI score were different between the groups (all *p* ≤ 0.001). The Bell’s palsy group showed a higher rate of obesity and higher CCI scores than the control group. The rate of alcohol consumption was higher in the Bell’s palsy group than in the control group.

**Figure 2:**
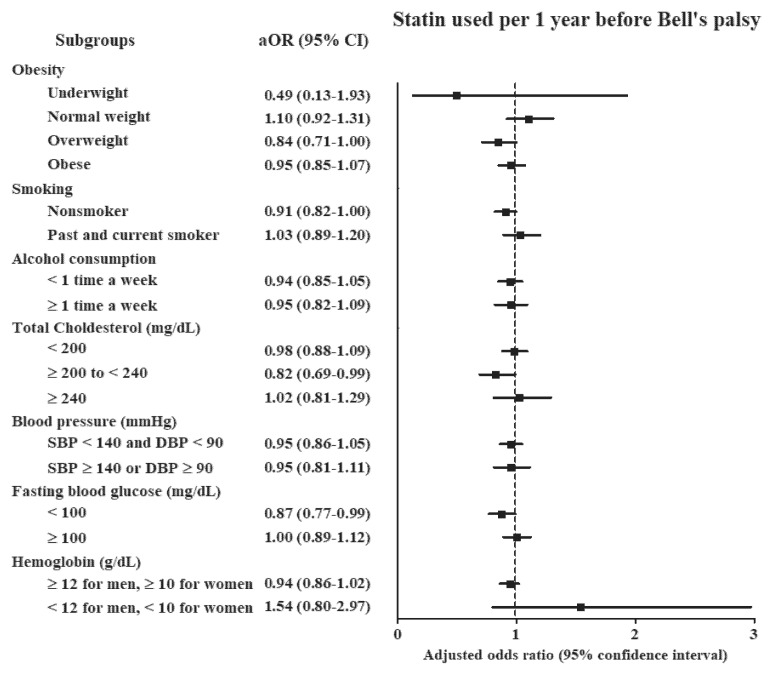
Odds ratios (95% confidence interval) of statin use against Bell’s palsy, according to age, sex, smoking, alcohol consumption, total cholesterol, blood pressure, fasting blood glucose, and hemoglobin.

The Bell’s palsy group demonstrated 1.11 times higher odds of previous statin use in the crude model (95% CI = 1.03–1.19, *p* = 0.006, Table 2). However, previous statin use was not related to Bell’s palsy in models 1 and 2. None of the age or sex subgroups demonstrated an association between Bell’s palsy and previous statin use in either model 1 or model 2. The ≥200 to <240 total cholesterol and <100 fasting blood glucose subgroups demonstrated lower odds of previous statin use in the Bell’s palsy group in model 2 than in the other models (adjusted OR = 0.82, 95% CI = 0.69–0.99, *p* = 0.038 for ≥200 to <240 total cholesterol and adjusted OR = 0.87, 95% CI = 0.77–0.99, *p* = 0.037 for <100 fasting blood glucose; Figure 2 and Table S1). No other subgroup analyses demonstrated any relationship between Bell’s palsy and previous statin use in either model 1 or model 2.

## 4. Discussion

We explored the relationship between previous statin use and Bell’s palsy in participants who had used statins for 2 years prior to the onset of the disease and found no relation between prior statin use and Bell’s palsy.

The results of the present study were consistent across various subgroups. We adjusted the models for laboratory measures and medical history based on health claim codes. In addition, the statin use history of participants during the previous 2 years was investigated to incorporate the potential residual effects of statins. This study helps to fill a gap in the existing research by demonstrating the associations between 2 years of statin use and Bell’s palsy in various subgroup analyses. This is the second and largest study evaluating the relation between statin use and Bell’s palsy.

A previous health claim cohort study reported the relationship between 6 months of statin use and Bell’s palsy [24], in which Bell’s palsy was defined based on its diagnostic codes (ICD-9-Clinical Modification code 351.0). However, Bell’s palsy is an exclusion diagnosis, and there are other organic causes of facial palsy such as brain trauma or tumor. In addition, the effects of previous statin use could last for approximately 2 years [30]. Because statins have been reported to have pleotropic effects against cardiovascular, neuronal, and viral infections [2,31,32], other medical history besides cardiovascular diseases needs to be adjusted for assessing the correlation between statin use and Bell’s palsy. The present study showed improved findings over previous research, owing to the exclusion of differential diagnoses for Bell’s palsy, a longer assessment period for previous statin use, and adjustments for comorbidities using CCI scores and laboratory measurements. Our results indicate no definite hazardous effect of previous statin use on Bell’s palsy.

Lipid-lowering and other pleiotropic effects could mediate the protective effects of statin use on Bell’s palsy. The neuroprotective effect of statins is exerted independently, different from the lipid-lowering effect [13,33]. Rosuvastatin recovered the vascularity of the vasa nervorum and nerve conduction velocities in diabetic neuropathy mice [33]. It was suggested that the restoration of neuronal nitric oxide synthase expression and the phosphorylation of Akt in Schwann cells were associated with the neuroprotective effects of rosuvastatin [33]. Several previous studies reported the anti-inflammatory and anti-oxidative effects of statins [34,35]. In the Danish General Suburban Population Study that enrolled 19,795 participants, statin users showed lower levels of markers for oxidative damage and chronic inflammation than non-users [34]. In addition, a meta-analysis study demonstrated a decrease in C-reactive protein, tumor necrosis factor-α, interleukin-6, and interleukin-1 levels in statin users with metabolic syndrome and related diseases [35]. Because Bell’s palsy is a peripheral neuropathy proposed to have inflammatory features [36], the neuroprotective and anti-inflammatory effects of statins could reduce the risk of Bell’s palsy.

However, some previous studies have demonstrated the neurotoxic effects of statins, for example, an increased risk of Bell’s palsy. Axonal degeneration and segmental demyelination were suggested to be related to neuropathy following statin use [37]. Statins inhibit the synthesis of ubiquinone, which is essential for the mitochondrial respiratory chain, thereby impeding energy supply to neurons [37]. Moreover, changes in neuronal cell membrane composition were suggested to impact protein interactions and neuronal functions [38]. A few previous case series and epidemiological studies reported peripheral neuropathy associated with statin use [15,39]. Nonetheless, the adverse effect of statins on neuropathy was as low as 1 person/14,000 person-years of treatment [15]. Furthermore, the ranges of ORs were wide, and with the heterogeneous study design, potential confounding factors could not be excluded (OR: 0.66–14.2, CI: 0.3–38) [40]. Thus, the adverse effects of statins on Bell’s palsy might be trivial, and statistical significance could not be established.

The Bell’s palsy group was matched with a non-Bell’s palsy group in this study. Total cholesterol level, blood pressure, blood glucose, hemoglobin, obesity, smoking, alcohol consumption, CCI score, and dyslipidemia were adjusted for analyzing the relationship between statin use and Bell’s palsy. To exclude other causes of facial palsy, patients with a history of trauma or brain tumor were excluded. In addition, comprehensive subgroup analyses were conducted.

Regardless, there are certain limitations to the present study, primarily owing to the lack of linked information in the cohort. First, because statin use was examined using prescription data, drug compliance could not be accounted for in this study. Second, information on statin type could not be obtained. The impact of statin use on Bell’s palsy might vary according to the types of statins used. The use of lipophilic statins is more closely associated with peripheral neuropathy than the use of less lipophilic statins [37]. Third, the severity and prognosis of Bell’s palsy could not be specified in the inclusion criteria of the Bell’s palsy group. Nevertheless, the results of this study might provide additional information that can prevent the unnecessary withdrawal of statins. 

## 5. Conclusions

We found that previous statin use was not associated with Bell’s palsy in this population ≥40 years old.

## Figures and Tables

**Table 1:** General characteristics of participants.

Characteristics	Total Participants		
	Bell’s Palsy	Control	*p*-Value
Age (years old, *n*, %)			1.000
40–44	22 (0.7)	88 (0.7)	
45–49	278 (8.7)	1112 (8.7)	
50–54	585 (18.3)	2340 (18.3)	
55–59	666 (20.8)	2664 (20.8)	
60–64	535 (16.7)	2140 (16.7)	
65–69	482 (15.1)	1928 (15.1)	
70–74	348 (10.9)	1392 (10.9)	
75–79	197 (6.2)	788 (6.2)	
80–84	76 (2.4)	304 (2.4)	
85+	14 (0.4)	56 (0.4)	
Sex (*n*, %)			1.000
Male	1675 (52.3)	6700 (52.3)	
Female	1528 (47.7)	6112 (47.7)	
Income (*n*, %)			1.000
1 (lowest)	472 (14.7)	1888 (14.7)	
2	406 (12.7)	1624 (12.7)	
3	500 (15.6)	2000 (15.6)	
4	709 (22.1)	2836 (22.1)	
5 (highest)	1116 (34.8)	4464 (34.8)	
Region of residence (*n*, %)			1.000
Urban	1423 (44.4)	5692 (44.4)	
Rural	1780 (55.6)	7120 (55.6)	
Total cholesterol (mg/dL, mean, SD)	198.5 (38.3)	199.5 (39.0)	0.229
SBP (mmHg, mean, SD)	127.7 (16.4)	126.5 (16.1)	<0.001 ^†^
DBP (mmHg, mean, SD)	79.0 (10.7)	78.2 (10.3)	<0.001 ^†^
Blood glucose (mg/dL, mean, SD)	104.7 (31.3)	101.5 (31.8)	<0.001 ^†^
Hemoglobin (g/dL, mean, SD)	13.9 (1.5)	13.8 (1.5)	0.009 ^†^
Obesity (*n*, %) ^‡^			<0.001 *
Underweight	38 (1.2)	286 (2.2)	
Normal	880 (27.5)	4480 (35.0)	
Overweight	897 (28.0)	3529 (27.5)	
Obese I	1238 (38.7)	4143 (32.3)	
Obese II	150 (4.7)	374 (2.9)	
Smoking status (*n*, %)			0.290
Nonsmoker	2232 (69.7)	8803 (68.7)	
Past smoker	444 (13.9)	1750 (13.7)	
Current smoker	527 (16.5)	2259 (17.6)	
Alcohol consumption (*n*, %)			0.001 *
<1 time a week	2166 (67.6)	8248 (64.4)	
≥1 time a week	1037 (32.4)	4564 (35.6)	
CCI score (score, *n*, %)			<0.001 *
0	2034 (63.5)	9077 (70.9)	
1	570 (17.8)	1699 (13.3)	
2	262 (8.2)	931 (7.3)	
3	154 (4.8)	496 (3.9)	
≥4	183 (5.7)	609 (4.8)	
Dyslipidemia (*n*, %)	1112 (34.7)	3794 (29.6)	<0.001 *
The day of statin used (day, mean, SD)	84.6 (201.7)	74.4 (189.4)	0.009 ^†^

Abbreviations: CCI, Charlson comorbidity index; DBP, diastolic blood pressure; SBP, systolic blood pressure; SD, standard deviation. * Chi-square test. Significance at *p* < 0.05; ^†^ Independent *t* test. Significance at *p* < 0.05; ^‡^ Obesity (BMI, body mass index, kg/m^2^) was categorized as <18.5 (underweight), ≥18.5 to <23 (normal), ≥23 to <25 (overweight), ≥25 to <30 (obese I), and ≥30 (obese II).

**Table 2:** Odds ratios (95% confidence interval) for the day of statin use per 1 year in the Bell’s palsy group compared to control group with subgroup analyses according to age and sex.

Characteristics	Odds Ratios					
	Crude ^†^	*p*-Value	Model 1 ^†,‡^	*p*-Value	Model 2 ^†,§^	*p*-Value
Total participants (*n* = 16,015)						
Statin use (per 1 year)	1.11 (1.03–1.19)	0.006 *	0.98 (0.90–1.06)	0.548	0.95 (0.87–1.03)	0.218
Age <60 years old, men (*n* =4510)						
Statin use (per 1 year)	1.15 (0.97–1.36)	0.102	1.01 (0.83–1.23)	0.919	0.97 (0.80–1.18)	0.750
Age <60 years old, women (*n* =3245)						
Statin use (per 1 year)	1.25 (1.01–1.55)	0.045 *	0.98 (0.76–1.25)	0.841	0.93 (0.72–1.20)	0.573
Age ≥60 years old, men (*n* = 3865)						
Statin use (per 1 year)	1.07 (0.93–1.22)	0.348	0.96 (0.82–1.12)	0.583	0.95 (0.81–1.11)	0.517
Age ≥60 years old, women (*n* = 4395)						
Statin use (per 1 year)	1.09 (0.97–1.22)	0.163	0.97 (0.85–1.11)	0.662	0.95 (0.83–1.09)	0.482

Abbreviations: CCI, Charlson comorbidity index; DBP, diastolic blood pressure; SBP, systolic blood pressure; * Conditional logistic regression, Significance at *p* < 0.05; ^†^ Models were stratified by age, sex, income, and region of residence. ^‡^ Model 1 was adjusted for dyslipidemia history, total cholesterol, SBP, DBP, blood glucose, and hemoglobin. ^§^ Model 2 was adjusted for dyslipidemia history, total cholesterol, SBP, DBP, blood glucose, hemoglobin, obesity, smoking, alcohol consumption, and CCI scores.

## Supplementary Materials

The following are available online at https://www.mdpi.com/1660-4601/17/22/8408/s1, S1: Description Study Population and Data Collection, Table S1: Subgroup analyses of odds ratios (95% confidence interval) for the day of statin used per 1 year in Bell’s palsy group compared to control group according to obesity, smoking, alcohol consumption, total cholesterol, blood pressure, blood glucose, and hemoglobin.
