# Supplementary material for: Relationship between Bell’s Palsy and Previous Statin Use: A Case/Non-Case Study
Source: Int J Environ Res Public Health. 2020 Nov 13;17(22):8408. doi: 10.3390/ijerph17228408 (PMC7696239; doi:10.3390/ijerph17228408)
Supplement: Supplementary file 1 [file ijerph-17-08408-s001.zip › ijerph-987369-supplementary.docx]

Supplementary material

Relationship between Bell’s Palsy and Previous Statin Use: A Case/Non-Case Study

So Young Kim ^1^, Jee Hye Wee ^2^, Chanyang Min ^3,4^, Dae-Myoung Yoo ^3^ and Hyo Geun Choi ^2,3,^*

^1^ Department of Otorhinolaryngology-Head & Neck Surgery, CHA Bundang Medical Center, CHA University, Seongnam 13496, Korea; sossi81@hanmail.net

^2^ Department of Otorhinolaryngology-Head & Neck Surgery, Hallym University College of Medicine, 22, Gwanpyeong-ro 170beon-gil, Dongan-gu, Anyang-si, Gyeonggi-do, Anyang 14068, Korea; weejh07@hanmail.net

^3^ Hallym Data Science Laboratory, Hallym University College of Medicine, Anyang, 14068, Korea; joicemin@naver.com (C.M.); ydm1285@naver.com (D.-M.Y.)

^4^ Graduate School of Public Health, Seoul National University, Seoul 08826, Korea

***** Correspondence: pupen@naver.com; Tel.: +82-31-380-3849

Received: 19 October 2020; Accepted: 11 November 2020; Published: date

S1. Description Study Population and Data Collection.

The data from the Korean National Health Insurance Service-Health Screening Cohort (NHIS-HEALS) was used.^1^ The Korean National Health Insurance Service (NHIS) chooses ~ 10% of random samples (n = ~515,000) directly from all people who had a health check-up from 2002 through 2003 year (n = ~5,150,000). The age and sex specific distributions of the cohort population are described in online.^2,3^

All of ≥ 40 years old Koreans and their families are requested to have a biannual health check without cost.^4^ Because all Korean citizens are registered with a 13-digit resident registration number for lifelong, the thorough population statistics can be calculated in this study. All Koreans have to register in the NHIS. The 13-digit resident registration number have to be used in all Korean hospitals and clinics. Thus, the medical records was prevented to be overlapped, even in case of a patient moves from one place to another. In addition, the Korean Health Insurance Review and Assessment (HIRA) system managed all medical treatments in Korea. The causes and date of death diagnosed by medical doctors on the death certificate are legally announced to administrative entity.

This NHIS included health insurance claim codes (procedures and prescriptions), diagnostic codes using the International Classification of Disease-10 (ICD-10), death records, socio-economic data and health check-up data (body mass index [BMI], drinking, smoking habit, blood pressure, urinalysis, hemoglobin, fasting glucose, lipid parameters, creatinine, and liver enzymes) for each participant over the period from 2002 to 2013.^3,4^

Covariates

Tobacco smoking was asked to the participant. Nonsmoker was who never smoked entire life. Past smoker was who quitted smoking currently. Current smoker is who are smoking currently. Alcohol consumption was categorized by the frequency of consumption (nondrinker or < 1 times a week, and 1-2 times a week, 3-4 times a week, almost every day.) This was re-categorized as < 1 time a week, and ≥ 1 time a week. Obesity was measured by BMI (body mass index, kg/m^2^). It was categorized as < 18.5 (underweight), ≥ 18.5 to < 23 (normal), ≥ 23 to < 25 (overweight), ≥ 25 to < 30 (obese I), and ≥ 30 (obese II) following the World Health Organization Western Pacific Regional Office (WHO-WPRO) 2000 classification

References

1 Lee, J., Lee, J. S., Park, S. H., Shin, S. A. & Kim, K. Cohort Profile: The National Health Insurance Service-National Sample Cohort (NHIS-NSC), South Korea. *International journal of epidemiology* **46**, e15, doi:10.1093/ije/dyv319 (2017).

2 Varini, M. *et al.* Hypereosinophilia in a boy with asthma and Varicella Zoster Virus infection. *Acta Biomed* **85**, 64-67 (2014).

3 http://nhiss.nhis.or.kr/.

4 Song, S. O. *et al.* Background and data configuration process of a nationwide population-based study using the korean national health insurance system. *Diabetes Metab J* **38**, 395-403, doi:10.4093/dmj.2014.38.5.395 (2014).

**Table S2.** Subgroup analyses of odds ratios (95% confidence interval) for the day of statin used per 1 year in Bell’s palsy group compared to control group according to obesity, smoking, alcohol consumption, total cholesterol, blood pressure, blood glucose, and hemoglobin.

| **Characteristics** | | | **Odds Ratios** | | | | | |
| --- | --- | --- | --- | --- | --- | --- | --- | --- |
|  |  |  | **Model ^1,†^** | **P-value** | **Model ^2,‡^** | ***p*-Value** | **Model ^3,§^** | ***p*-Value** |
| Obesity | | | | | | | | |
|  | Underweight (n = 324) | | | | | | | |
|  |  | Statin used (per 1 year) | 0.75 (0.22–2.53) | 0.644 | 0.48 (0.12–1.87) | 0.288 | 0.49 (0.13–1.93) | 0.309 |
|  | Normal weight (n = 5360) | | | | | | | |
|  |  | Statin used (per 1 year) | 1.18 (1.01–1.37) | 0.039 * | 1.11 (0.93–1.33) | 0.256 | 1.10 (0.92–1.31) | 0.310 |
|  | Overweight (n = 4426) | | | | | | | |
|  |  | Statin used (per 1 year) | 0.98 (0.84–1.14) | 0.794 | 0.84 (0.71–1.00) | 0.050 | 0.84 (0.71–1.00) | 0.052 |
|  | Obese (n = 5905) | | | | | | | |
|  |  | Statin used (per 1 year) | 1.06 (0.96–1.17) | 0.242 | 0.95 (0.85–1.07) | 0.397 | 0.95 (0.85–1.07) | 0.399 |
| Smoking | | |  |  |  |  |  |  |
|  | Nonsmoker (n = 11,035) | |  |  |  |  |  |  |
|  |  | Statin used (per 1 year) | 1.09 (1.00–1.19) | 0.055 | 0.94 (0.85–1.03) | 0.192 | 0.91 (0.82–1.00) | 0.060 |
|  | Past smoker and current smoker (n = 4980) | |  |  |  |  |  |  |
|  |  | Statin used (per 1 year) | 1.15 (1.01–1.31) | 0.031 * | 1.07 (0.92–1.24) | 0.394 | 1.03 (0.89–1.20) | 0.677 |
| Alcohol consumption | | |  |  |  |  |  |  |
|  | <1 time a week (n = 10,414) | |  |  |  |  |  |  |
|  |  | Statin used (per 1 year) | 1.10 (1.01–1.21) | 0.030 * | 0.97 (0.88–1.08) | 0.611 | 0.94 (0.85–1.05) | 0.277 |
|  | ≥1 time a week (n = 5601) | |  |  |  |  |  |  |
|  |  | Statin used (per 1 year) | 1.13 (1.00–1.28) | 0.057 | 0.98 (0.85–1.13) | 0.747 | 0.95 (0.82–1.09) | 0.441 |
| Total cholesterol (mg/dL) | | |  |  |  |  |  |  |
|  | <200 (n = 8423) | |  |  |  |  |  |  |
|  |  | Statin used (per 1 year) | 1.15 (1.06–1.26) | 0.002 * | 0.99 (0.89–1.11) | 0.907 | 0.98 (0.88–1.09) | 0.640 |
|  | ≥200 to <240 (n = 5364) | |  |  |  |  |  |  |
|  |  | Statin used (per 1 year) | 0.98 (0.83–1.15) | 0.813 | 0.84 (0.70–1.01) | 0.064 | 0.82 (0.69–0.99) | 0.038 * |
|  | ≥240 (n = 2228) | |  |  |  |  |  |  |
|  |  | Statin used (per 1 year) | 1.07 (0.87–1.31) | 0.521 | 1.06 (0.85–1.33) | 0.607 | 1.02 (0.81–1.29) | 0.859 |
| Blood pressure (mmHg) | | |  |  |  |  |  |  |
|  | SBP <140 and DBP <90 (n = 12,051) | |  |  |  |  |  |  |
|  |  | Statin used (per 1 year) | 1.13 (1.04–1.23) | 0.005 * | 0.97 (0.88–1.08) | 0.598 | 0.95 (0.86–1.05) | 0.286 |
|  | SBP ≥140 or DBP ≥90 (n = 3964) | |  |  |  |  |  |  |
|  |  | Statin used (per 1 year) | 1.04 (0.91–1.20) | 0.537 | 0.97 (0.83–1.14) | 0.730 | 0.95 (0.81–1.11) | 0.535 |
| Fasting blood glucose (mg/dL) | | |  |  |  |  |  |  |
|  | <100 (n = 9717) | |  |  |  |  |  |  |
|  |  | Statin used (per 1 year) | 1.05 (0.94–1.17) | 0.377 | 0.89 (0.79–1.01) | 0.073 | 0.87 (0.77–0.99) | 0.037 * |
|  | ≥100 (n = 6298) | |  |  |  |  |  |  |
|  |  | Statin used (per 1 year) | 1.11 (1.01–1.23) | 0.032 * | 1.03 (0.92–1.16) | 0.577 | 1.00 (0.89–1.12) | 0.938 |
| Hemoglobin (g/dL) | | |  |  |  |  |  |  |
|  | ≥12 for men and ≥ 10 for women  (n = 15,736) | |  |  |  |  |  |  |
|  |  | Statin used (per 1 year) | 1.10 (1.02–1.18) | 0.010 * | 0.96 (0.88–1.05) | 0.363 | 0.94 (0.86–1.02) | 0.135 |
|  | <12 for men and <10 for women (n = 279) | |  |  |  |  |  |  |
|  |  | Statin used (per 1 year) | 1.38 (0.80–2.37) | 0.249 | 1.76 (0.94–3.30) | 0.078 | 1.54 (0.80–2.97) | 0.200 |

Abbreviations: CCI, Charlson comorbidity index; DBP, diastolic blood pressure; SBP, systolic blood pressure. * Logistic regression, Significance at *p* < 0.05. ^†^ A model 1 was adjusted for age, sex, income, and region of residence. ^‡^ A model 2 was adjusted for age, sex, income, region of residence, dyslipidemia history, total cholesterol, SBP, DBP, blood glucose, and hemoglobin. ^§^ A model 3 was adjusted for age, sex, income, and region of residence, dyslipidemia history, total cholesterol, SBP, DBP, blood glucose, hemoglobin, obesity, smoking, alcohol consumption, and CCI scores (subgroup analyses according to obesity did not include obesity as a covariate. subgroup analyses according to smoking did not include smoking status as a covariate. subgroup analyses according to alcohol consumption did not include alcohol consumption as a covariate.).
